# The adaptor protein 14-3-3zeta modulates intestinal immunity and aging in *Drosophila*

**DOI:** 10.1016/j.jbc.2023.105414

**Published:** 2023-10-31

**Authors:** Xiaolan Fan, Tiantian Huang, Shuai Wang, Ziyue Yang, Wenhao Song, Yao Zeng, Yingdong Tong, Yujuan Cai, Deying Yang, Bo Zeng, Mingwang Zhang, Qingyong Ni, Yan Li, Diyan Li, Mingyao Yang

**Affiliations:** 1 Institute of Animal Genetics and Breeding, Sichuan Agricultural University, Chengdu, Sichuan, P. R. China; 2 Farm Animal Genetic Resources Exploration and Innovation Key Laboratory of Sichuan Province, Sichuan Agricultural University, Chengdu, Sichuan, China; 3 Technology Institute of Silk and Mulberry, Chong Qing Academy of Animal Sciences, Chongqing, P. R. China

**Keywords:** 14-3-3zeta, lifespan, intestinal immunity, aging, *Drosophila*

## Abstract

The proteins that coordinate the complex transcriptional networks of aging have not been completely documented. Protein 14-3-3zeta is an adaptor protein that coordinates signaling and transcription factor networks, but its function in aging is not fully understood. Here, we showed that the protein expression of 14-3-3zeta gradually increased during aging. High levels of 14-3-3zeta led to shortened lifespan and imbalance of intestinal immune homeostasis in *Drosophila*, but the decrease in 14-3-3zeta protein levels by RNAi was able to significantly promote the longevity and intestinal immune homeostasis of fruit flies. Importantly, we demonstrate that adult-onset administration of TIC10, a compound that reduces the aging-related AKT and extracellular signal-regulated kinase (ERK) signaling pathways, rescues the shortened lifespan of 14-3-3zeta-overexpressing flies. This funding suggests that 14-3-3zeta plays a critical role in regulating the aging process. Our study elucidates the role of 14-3-3zeta in natural aging and provides the rationale for subsequent 14-3-3zeta-based antiaging research.

Aging is characterized by a progressive deterioration in physiological integrity, resulting in impaired functional capacity and ultimately increased susceptibility to death. Biological aging is accompanied by chronic low-grade inflammation levels, a chronic phenomenon known as inflammatory aging, and a very important risk factor for morbidity and mortality in the elderly (1, 2). Major chronic diseases (cardiovascular disease, cancer, type 2 diabetes, sarcopenia, osteoporosis, Alzheimer’s disease, and frailty) are associated with inflammation and aging, which share similar inflammatory pathogenesis (3). Therefore, investigating the relationship between aging and chronic inflammation can provide new strategies for treating these age-related diseases.

The 14-3-3 proteins are a family of conserved regulatory adaptor molecules that are expressed in all eukaryotic cells (4, 5, 6). Moreover, these proteins act as molecular chaperones, preventing the aggregation of unfolded proteins under conditions of cellular stress (7). Only two isoforms (zeta and epsilon) have been reported in *Drosophila* and *Caenorhabditis elegans* (4). In mammals, 14-3-3 proteins are highly conserved and consist of seven isoforms (β, γ, ζ, η, θ, σ, and ε) with unique expression patterns in different cell types and tissues (8). The 14-3-3 proteins exist in the form of a homodimer or heterodimer and can have a rigid structure, in which two phosphopeptide binding sites can work as adaptors or allosteric regulators of their targets (9). These targets include kinases and proteins involved in DNA replication, apoptosis, metabolism, the cell cycle, aging, and the response to DNA damage (10, 11, 12).

The 14-3-3s interact with translationally controlled tumor protein (Tctp) and Rheb GTPase to regulate organ growth in *Drosophila* (13). Recent studies have shown that Tctp and 14-3-3epsilon play critical roles in cell growth by reducing cytoplasmic FOXO levels, while 14-3-3zeta plays a lesser role in this process, suggesting an isoform-specific role of 14-3-3s (14). In addition, *Drosophila* 14-3-3epsilon is also involved in longevity as a central regulator of FOXO activity (15). Earlier studies have shown that *Drosophila* 14-3-3zeta is a heat-induced molecular chaperone that prevents and reverses heat-induced protein aggregation (16). As the adapter protein, 14-3-3zeta regulates numerous signal transduction pathways, including GM-CSF, IL-3, IL-5, STAT3, Toll-like receptor (TLR)-3, and AMPK (17, 18), and participates in the development, metabolism, protein degradation and cancer processes (7, 19). More than 100 target proteins of 14-3-3zeta have been identified, including phosphorylated TSC2 for the inhibition of p70S6 kinase activity (20) and PI3K for the activation of PI3K/AKT signaling (21). The 14-3-3zeta acts as a negative regulator of autophagy by inhibiting hVps34 kinase activity, which is essential for autophagy membrane assembly in the early stage of autophagy (22, 23). Although these proteins or processes play an important role in aging, the network and function of 14-3-3zeta in the regulation of aging have not yet been fully established.

The AKT/FOXO axis and Ras/ERK axis play key roles in the regulation of diverse processes ranging from autophagy, DNA damage responses, and cellular senescence to aging (24). *akt* and *ras* mutant flies have extended lifespans, while inhibitors of these two axes have been shown to have lifespan-extending effects (25, 26). TIC10, also known as ONC201, inactivates the kinases AKT and ERK (27). Currently, TIC10 has been confirmed to have activity against a variety of tumors (28, 29, 30). Mechanistic investigation showed that mitochondrial caseinolytic protease P (ClpP) is the target of TIC10 (31, 32, 33). However, there is no report on whether TIC10 is involved in the regulation of aging and the promotion of longevity. Here, we present molecular evidence of 14-3-3zeta in the control of *Drosophila* lifespan.

## Results

### The expression level of 14-3-3 zeta increased with aging

**Figure 1:**
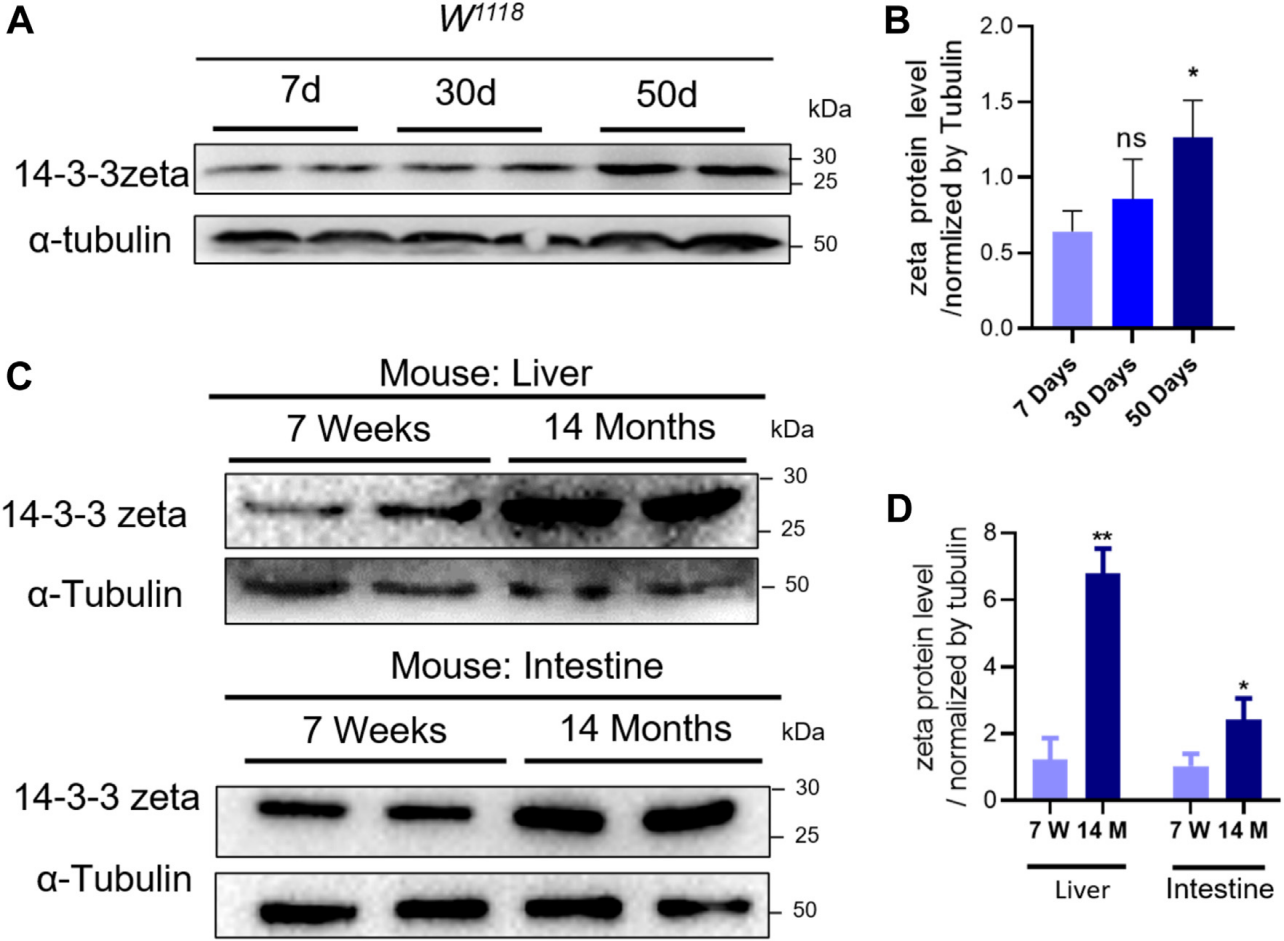
**The 14-3-3 protein level increased during the aging.***A*, the 14-3-3 protein level in different age of *Drosophila*. *B*, the bar graph illustrated the densitometry of the blots (ns: *p* > 0.05, ∗*p* < 0.05, n = 2 means 60 flies pooled from 2 independent experiments). *C*, the 14-3-3 protein level in different age of mouse liver and intestine tissue. *D*, the bar graph illustrated the densitometry of the blots (∗*p* < 0.05, ∗∗*p* < 0.01, n = 2 means 6 mice pooled from 2 independent experiments).

The 14-3-3 adapter protein family regulates multiple processes of the cell. The 14-3-3zeta isoform regulates diverse cellular functions, including cell proliferation, metabolism, adhesion, and apoptosis (34). To evaluate whether 14-3-3zeta participates in the aging process, we first assessed the protein expression level of 14-3-3zeta in wild-type flies on Days 7, 30, and 50. The expression level of 14-3-3zeta significantly increased from d7 to d50 (Fig. 1, *A* and *B*). Meanwhile, we examined the protein of 14-3-3zeta in the livers and intestines of young (7 weeks) and old (14 months) mice and found that its level was also significantly increased in 14-month-old mice (Fig. 1, *C* and *D*). These data indicate that 14-3-3zeta had a high expression level in the aged animals.

### 14-3-3zeta regulates *Drosophila* aging *in vivo*

This study observed an increase in protein levels of 14-3-3zeta with aging in both *Drosophila* and mice, indicating a potential broader role of 14-3-3zeta in the aging process. To further investigate this hypothesis, we conducted experiments in *Drosophila.* First, we recruited the gene-switch GAL4 expression system and used RU486 to induce the overexpression of the *14-3-3zeta* in *Drosophila* adults. To observe the effect of RU on the 14-3-3zeta protein level, we detected the 14-3-3zeta protein in *Da*^*GS*^*gal4>W*^*1118*^ flies (Fig. S1*A*). Treatment with RU486 did not change the protein level of 14-3-3zeta. In *Da*^*GS*^*gal4>zeta* flies, the addition of RU486 significantly increased the protein level of 14-3-3zeta (Fig. S1*B*), confirming that the gene-switch system worked well in our experiments.

**Figure 2:**
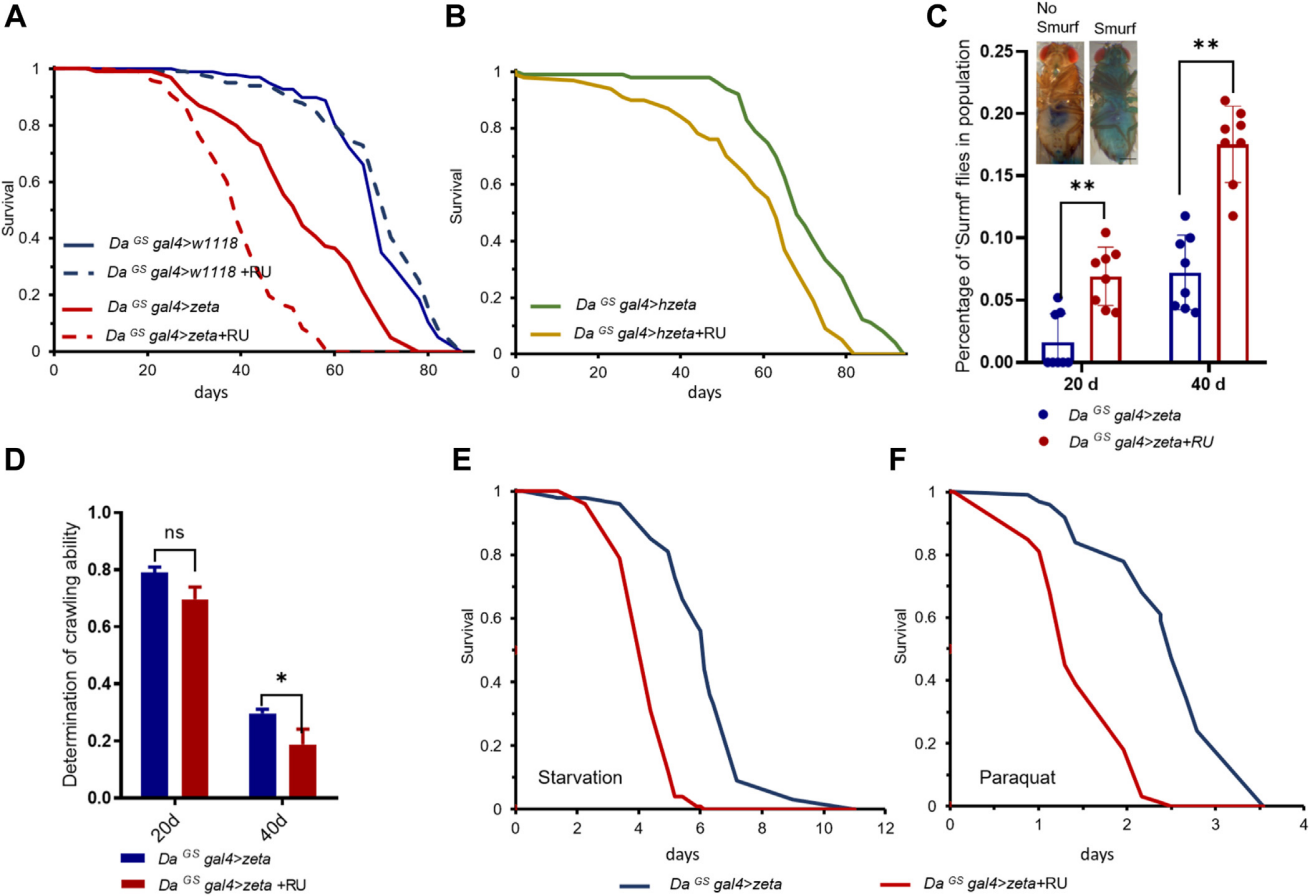
**14-3-3zeta regulates lifespan and aging.***A*, flies overexpressing 14-3-3zeta were collected and subjected to life longevity assays. Survival curves were analyzed (*p* < 0.0001, n = 100, log-rank test). *B*, lifespan of flies overexpressing human h14-3-3zeta using the ubiquitous Daughterless-Gal4 (da-Gal4) driver (*p* < 0.0001, n = 100, log-rank test). *C*, the percentage of ‘‘Smurf’’ flies with the indicated genotypes (∗∗*p* < 0.01, n > 90 females for each genotype, Scale bars: 500 μm). *D*, effect on the climbing ability of flies overexpressing 14-3-3zeta (ns *p* > 0.05, ∗*p* < 0.05, n > 90 females for each genotype). *E* and *F*, effect on tolerance to starvation and paraquat in flies (*p* < 0.0001, n = 100, log-rank test).

Regarding fly survival measurement, RU treatment with *Da*^*GS*^*gal4>zeta* reduced the fly lifespan, while the lifespan between *Da*^*GS*^*gal4>W*^*1118*^ treated or not treated with RU was not different (Fig. 2*A*). To further address whether this protein could regulate fly lifespan through specific tissues, we used the *S106*^*GS*^ *gal4*, which is specifically expressed in the fat body, and *5966*^*GS*^ *gal4*, which is expressed in the intestinal differentiated cells, to overexpress *14-3-3zeta* specifically in these tissues. The results showed that the specific overexpression of *14-3-3zeta* in fat bodies or intestinal cells also significantly reduced the fly lifespan (Fig. S1, *C* and *D*). The reduction in the lifespans of both tissue-specific overexpression lines was weaker than the reduction in the lifespan of the ubiquitous overexpression lines. Then, to investigate whether human 14-3-3zeta regulates the lifespan process, we constructed human *14-3-3zeta* into transgenic flies. The systemic overexpression of human *14-3-3zeta* in flies also significantly reduced their lifespan (Fig. 2*B*).

Gut intestinal barrier function is a marker for intestinal permeability (35, 36). Flies with intestinal integrity will limit the dye to the digestive tract, while *Drosophila* with intestinal integrity dysfunction will spread the dye throughout the body, such a fly is called "Smurf” (as the photo shown in Fig. 2*C*). The proportion of “Smurfs” in the *14-3-3zeta*-overexpressing population significantly increased when compared with that proportion in the control group from Day 20 to Day 40 (Fig. 2*C*). However, compared with controls of the same age, the climbing ability of the *14-3-3zeta* overexpressed group of flies was significantly reduced on Day 40 (Fig. 2*D*). Similarly, 1*4-3-3zeta*-overexpressing flies were significantly less able to respond to starvation stress (Fig. 2*E*) and paraquat-induced oxidative stress (Fig. 2*F*) than controls. These results demonstrated that the elevated level of 14-3-3zeta during aging was detrimental to maintaining a normal health span and lifespan.

### 14-3-3zeta participates in intestinal immune homeostasis during aging

**Figure 3:**
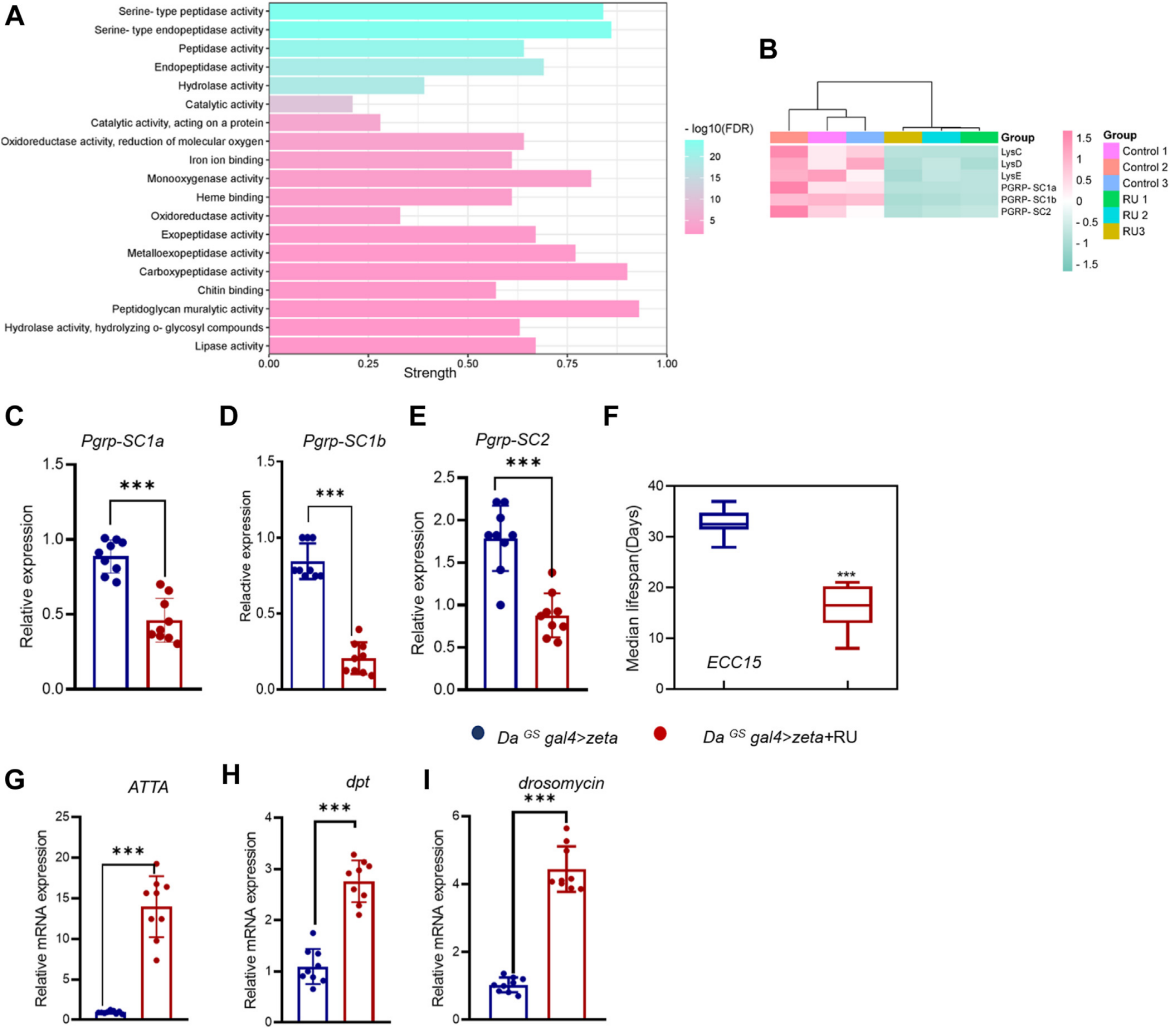
**Differentially expressed genes in *14-3-3zeta*-overexpressing flies.***A*, the molecular function by GO enrichment analysis of differentially expressed genes between control and 14-3-3zeta overexpression. *B*, heatmap of the genes that enriched in the “Peptidoglycan muralytic activity” function between control and *14-3-3zeta* overexpression groups. *C–E*, the RNA expression levels of the indicated genes in 14-3-3zeta-overexpressing flies (Error bars indicate SD, ∗*p* < 0.05, ∗∗*p* < 0.01, ∗∗∗*p* < 0.001, n = 3). *F*, the median lifespan of the indicated genotype and infected with ECC15. *G*–*I*, The RNA expression level of the indicated genes in the control and 14-3-3zeta-overexpressing flies (Error bars indicate SD, ∗*p* < 0.05, n = 3 means 30 flies pooled from 3 independent experiments).

To explore the molecular mechanisms underlying the effects of 14-3-3zeta on health span and lifespan, we performed RNA-seq analysis using age-matched samples from (30-day-old) female *Da*^*GS*^ *gal4>zeta* adult flies treated with or without RU. Fragments per kilobase of exon per million mapped fragments (FPKM) analysis indicated that the overall gene expression patterns were similar between the two experimental groups (Fig. S2*A*). However, further differential expression analysis identified a total of 349 differentially expressed genes, of which 24 were upregulated and 325 were downregulated (Fig. S2*B*). Interestingly, Gene Ontology analysis (Molecular Function) of these genes showed that the genes with peptidoglycan muralytic activity were greatly altered by overexpression of *14-3-3zeta* (Fig. 3*A*). The expression levels of these genes, including peptidoglycan-recognition proteins (PGRPs), *pgrp-sc1a*, *pgrp-sc1b*, *pgrp-sc2*, and lysozyme encoding genes, *lysC*, *lysD lysE* were significantly decreased in the 14-3-3zeta overexpression groups (Fig. 3*B*), compared with the control groups. To further confirm the results obtained from RNA-seq analysis, we carried out quantitative reverse transcription polymerase chain reaction (qRT-PCR) to quantify the relative expression levels of *pgrp-sc*s in *Drosophila* samples obtained at 30 days. As shown in (Fig. 3, *C*–*E*), the mRNA expression levels of genes, including *pgrp-sc1a*, *pgrp-sc1b*, and *pgrp-sc2*, were significantly decreased.

PGRP-SC has been reported to be the main negative regulator of IMD pathway activation in fat bodies (37). PGRP-SC2 has been reported to promote intestinal immune homeostasis and limit commensal dysbiosis, which can prolong the lifespan of *Drosophila* (38). The genes encoding lysozyme may have a function in the digestion of bacteria in food and are involved in intestinal pathogenic bacterial infection (39). Therefore, we paid particular attention to the state of the immune system of the overexpressed *14-3-3zeta* flies. When the gram-negative phytopathogenic bacterium *ECC15* was used to infect *Drosophila* orally, we found that overexpressing *14-3-3zeta* flies died within 25 days, with the lifespan significantly shortened compared with the lifespan of control flies (Fig. 3*F*). In addition, transcription levels of genes encoding immunodeficiency (IMD)-related AMPs, such as *diptericin* (*Dpt*), and *attacin* (*ATTA*), and the Toll-related AMP, *drosomycin* were significantly increased in the guts of flies overexpressing *14-3-3zeta*, compared with those in age-matched wild-type controls (Fig. 3, *H* and *I*). These results implied that the overexpression of *14-3-3zeta* altered the expression of immune-related genes in the guts.

### Reduced expression of 14-3-3zeta to promote longevity

**Figure 4:**
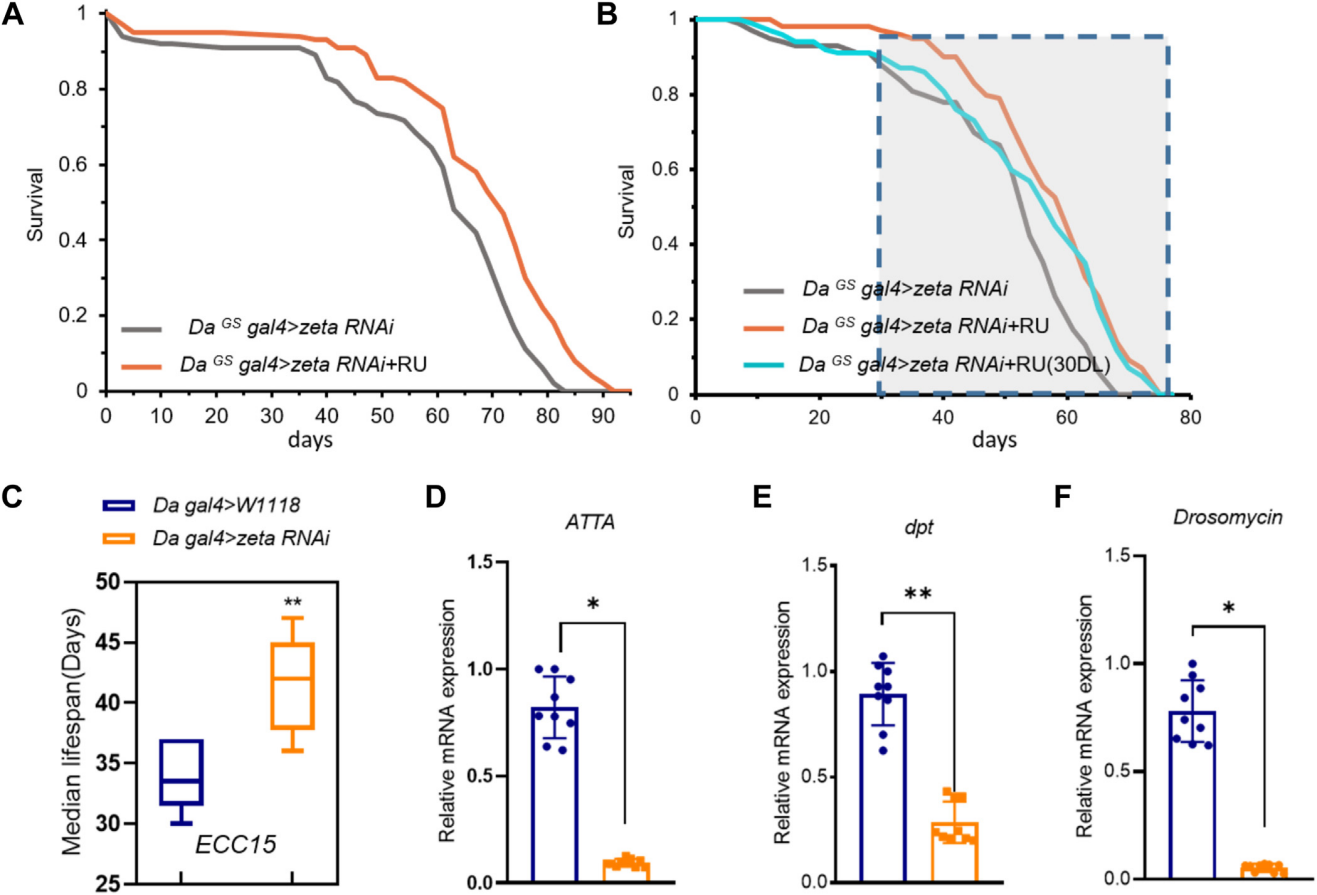
**Reduced 14-3-3zeta promoted longevity.***A*, ubiquitous knockdown of *14-3-3zeta* extended the lifespan (RNAi strains: *THU2964*), (*p* < 0.0001; log rank test, n = 100). *B*, flies by the ubiquitous knockdown of *14-3-3 zeta* for the lifetime or 30 days later after eclosion to the rest of the life (the shaded box marks the stage of silencing 14-3-3zeta), (RNAi *versus* control *p* < 0.001, RNAi *versus* 30 days later RNAi *p* > 0.05, n = 100 log-rank test). *C*, the median lifespan of the indicated genotype and infected with *ECC15* (∗∗*p* < 0.01, n = 100). *D–F*, the RNA expression levels of the indicated genes in *14-3-3zeta* knockdown flies (Error bars indicate SD, ns *p* > 0.05, ∗*p* < 0.05, ∗∗*p* < 0.01, n = 3).

To further confirm that 14-3-3zeta is the factor regulating lifespan, we speculated that the silencing of *14-3-3zeta* expression could also have an impact on longevity. By using two different *14-3-3zeta*-RNAi constructs (Fig. S3*A*), we found that the knockdown of *14-3-3zeta* significantly increased the fly lifespan, where the median lifespan was extended by 13.7% and 6.25%, respectively (Figs. 4*A* and S3*B*). As we had shown earlier the protein expression of 14-3-3zeta increased in middle age in flies (Fig. 1*A*), we tested whether *14-3-3zeta* was silenced after 30 days to resist the increase in 14-3-3zeta during aging. The results showed that the knockdown of *14-3-3zeta* after 30 days indeed prolonged the fly lifespan, and the median lifespan was extended by an average of 8.57% (Fig. 4*B*).

We also used *Da-gal4* to induce *14-3-3zeta* silencing (Fig. S3*C*) for *ECC15* infection experiments. When *ECC15* was used to infect flies, the median lifespan of the *14-3-3zeta* knockdown flies significantly increased compared with the lifespan of the control group (Fig. 4*C*). To explore the effect of 14-3-3zeta on the activity of immune regulatory pathways, we examined the expression levels of antimicrobial peptide genes in the *Drosophila* gut. As shown in Figure 4, *D*–*F*, transcription levels of *Dpt*, *ATTA*, and *drosomycin* were significantly decreased in guts from the *14-3-3zeta* knockdown flies, compared with the flies in age-matched wild-type controls.

These results confirm that reducing the levels of 14-3-3zeta protein during aging is beneficial for maintaining immune homeostasis and extending the lifespan of *Drosophila*.

### Overexpression of *14-3-3zeta* enhanced ERK and AKT signaling activity

**Figure 5:**
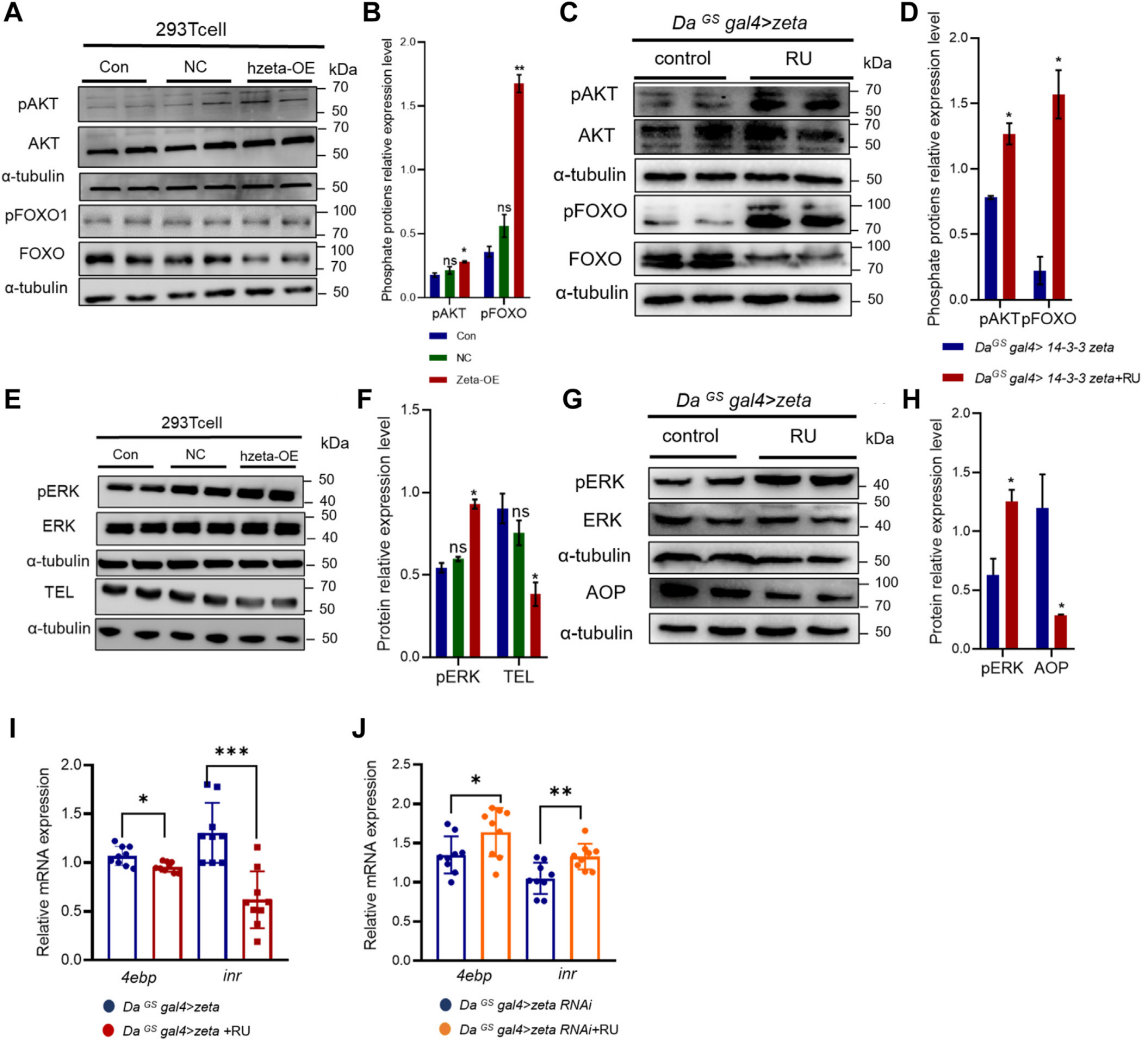
**The overexpression of 14-3-3zeta activated pAKT and pERK signaling.***A*, cultured cells were collected, and samples were then lysed and subjected to Western blotting to examine the levels of the indicated proteins. *B*, the bar graph illustrates the densitometry of the blots (con = control, NC = Negative control, hzeta-OE = human 14-3-3zeta overexpression, Error bars indicate SD, ns *p*>0.05, ∗*p*<0.05, ∗∗*p* < 0.01, n = 2 means 6 wells of cells pooled from 2 independent experiments). *C*, flies with the indicated genotypes at various ages were examined for the levels of the indicated proteins. *D*, the bar graph illustrates the densitometry of the blots (Error bars indicate SD, ∗*p* < 0.05, n = 2 means 60 flies pooled from 2 independent experiments). *E*, cultured cells were examined for the levels of the indicated proteins. *F*, the bar graph illustrates the densitometry of the blots (Error bars indicate SD, ∗*p* < 0.05, n = 2). *G*, flies with the indicated genotypes at various ages were examined for the levels of the indicated proteins. *H*, the bar graph illustrates the densitometry of the blots (Error bars indicate SD, ∗*p* < 0.05, n = 2). *I*, the RNA expression level of the indicated genes in the control and 14-3-3zeta-overexpressing flies (Error bars indicate SD, ∗*p* < 0.05, n = 3). *J*, The RNA expression levels of the indicated genes in *14-3-3zeta* knockdown flies (Error bars indicate SD, ns *p* > 0.05, ∗*p* < 0.05, ∗∗*p* < 0.01, n = 3).

The adaptor protein 14-3-3zeta can recognize phosphorylated FOXO by AKT (40). As we showed earlier that 14-3-3zeta plays a role in the regulation of *Drosophila* longevity, we explored the relationship between 14-3-3zeta and aging signaling. Previous results have shown that 14-3-3zeta is increased during natural aging (Fig. 1), so we mainly examined the effect of *14-3-3zeta* overexpression. Therefore, we tested the protein levels in the FOXO signaling pathway. pAKT is upstream of FOXO, which can inhibit the transcription factor FOXO by phosphorylation, preventing its nuclear translocation (41). Our results revealed that the phosphorylation (Ser473) of AKT was significantly increased in 293T cells overexpressing human *14-3-3zeta* (Figs. 5*A* and S4, *A* and *B*). Accordingly, the phosphorylation of FOXO (pFOXO) also increased significantly, while the protein level of unphosphorylated FOXO decreased significantly (Fig. 5*B*). Meanwhile, in *Drosophila,* we observed that when fly *14-3-3zeta* was overexpressed, the pAKT and pFOXO significantly increased, and unphosphorylated FOXO decreased (Fig. 5, *C* and *D*). These data suggested that the overexpression of *14-3-3zeta* can activate pAKT signaling.

The 14-3-3zeta regulates MAPK pathways (42), and MAPK signaling is involved in the regulation of *Drosophila* longevity (25). Therefore, we examined the phosphorylation of ERK (pERK) and anterior open (AOP) in MAPK pathways. The results demonstrated that the level of pERK (42/44) increased significantly in 293T cells overexpressing human 14-3-3zeta (Fig. 5, *E* and *F*). In contrast, the expression level of the transcription factor TEL (mammalian homology of AOP proteins) was reduced in the cells because its activity was inhibited by pERK. Similarly, in *Drosophila*, the protein of pERK increased, while the protein expression of AOP decreased significantly (Fig. 5, *G* and *H*), indicating that overexpression of *14-3-3zeta* both in the cell and fly can activate pERK signaling. In addition, to determine whether 14-3-3zeta exerts its effect through FOXO, we detected the regulation of FOXO target genes by 14-3-3zeta. We found that overexpression of *14-3-3zeta* significantly decreased the mRNA expression levels of FOXO target genes *inr* and *4ebp* (Fig. 5*I*). However, silencing of *14-3-3zeta* significantly increased the transcript expression levels of *inr* and *4ebp* (Fig. 5*J*). These results indicate that 14-3-3zeta inhibits FOXO and AOP protein levels in both *Drosophila* and humans.

### Inhibitor TIC10 restored the lifespan in the 14-3-3zeta-overexpressed fly

**Figure 6:**
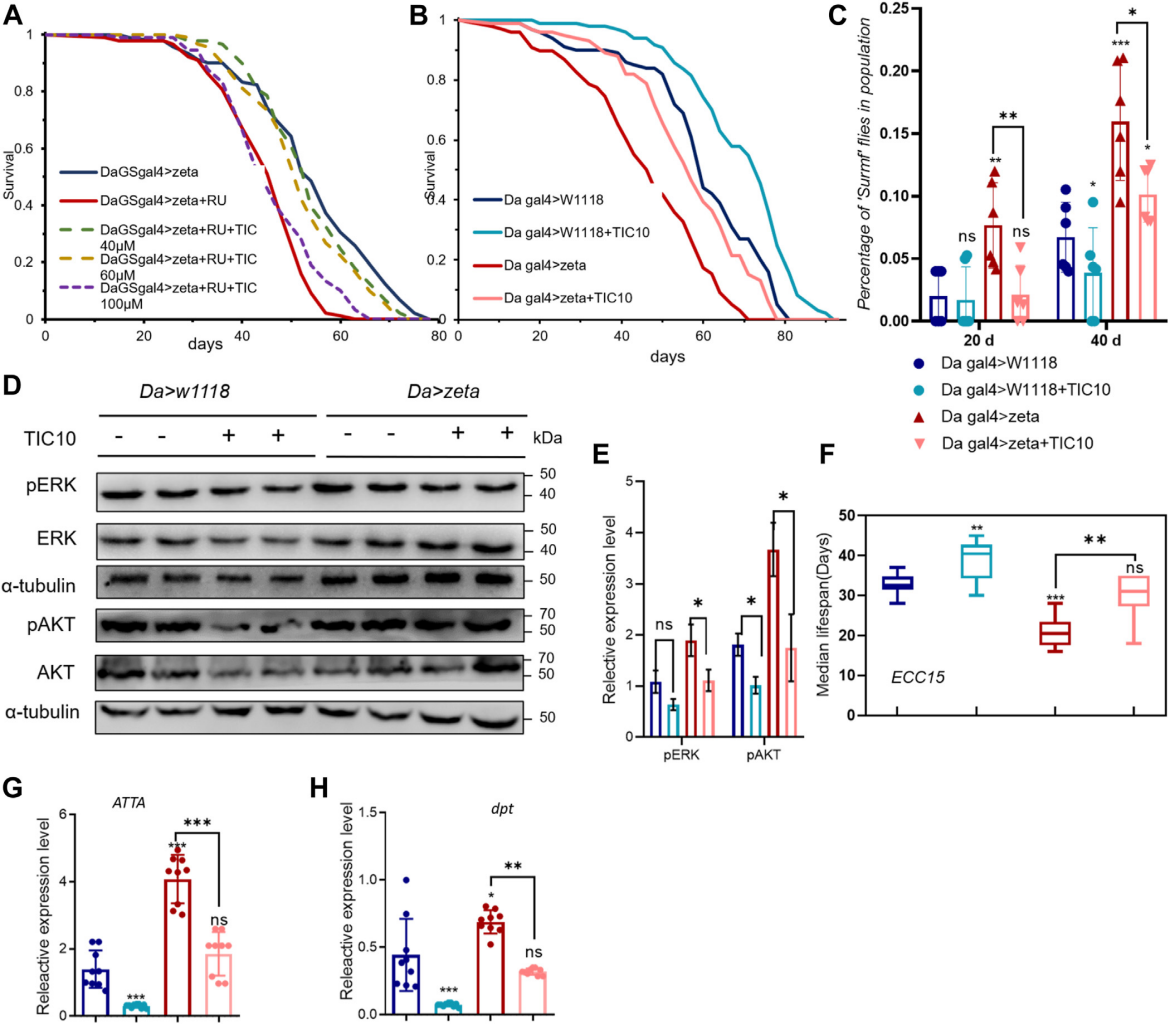
**TIC10 restored the aging phenotype caused by overexpression of *14-3-3zeta***. *A*, flies overexpressing 14-3-3zeta were treated with 40 ∼ 100 μM TIC10 and subjected to life longevity assays. Survival curves were analyzed (*p* < 0.0001, n = 100, log-rank test). *B*, flies with 14-3-3zeta overexpression treated with 40 μM TIC10 and subjected to life longevity assays. Da-gal4>W^1118^ was used as the control group, and survival curves were analyzed (*p* < 0.0001, n = 100, log-rank test). *C*, flies of different genotypes were subjected to ‘‘Smurf’’ assays. The proportion of flies showing loss of intestinal integrity was analyzed (Error bars indicate SD, ∗∗*p* < 0.01, n > 90). *D* and *E*, the protein level in flies with the indicated genotypes and treatment. The bar graph illustrated the densitometry of the blots (Error bars indicate SD, ∗*p* < 0.05, n = 2). *F*, the median lifespan of the indicated genotype and infected with *ECC15* (Significant differences on the bars are marked as comparisons with da-gal4>W^*1118*^, ns *p* > 0.05, ∗∗*p* < 0.01, ∗∗∗*p* < 0.001, n = 100). *G* and *H*, the RNA expression levels of the indicated genes in flies (Significant differences on the bars are marked as comparisons with da-gal4>W^*1118*^, ns *p* > 0.05, ∗∗∗*p* < 0.001, n = 3).

As shown above, 14-3-3zeta activates the AKT and ERK signaling pathways during aging. We therefore hypothesized that inhibition of these two pathways might rescue the shortened lifespan in *Drosophila* overexpressing *14-4-3zeta*. We then recruited TIC10, a drug that causes the dephosphorylation and inactivation of AKT and MEK in athymic nude mice (27), to investigate its positive role in *Drosophila*. A range of doses (40 μM, 60 μM and 100 μM) of TIC10 were administered to adult flies by supplementation in a food medium. The results showed that the shortened lifespans caused by overexpressed zeta (Fig. S5*A*) could be rescued at concentrations of 40 μM and 60 μM TIC10 (median lifespans from 46 to 53 and 51 days, respectively). However, survival rescue did not emerge in the group treated with 100 μm TIC10 (Fig. 6*A*). More interestingly, TIC10, which reduces both AKT and ERK activity, has a better rescue effect on shortening lifespan due to *14-3-3zeta* overexpression than trametinib, an inhibitor of ERK (Fig. S5*B*), suggesting that 14-3-3zeta plays a role in regulating lifespan through both AKT and ERK.

To eliminate the possible effect of RU486 on the efficacy of TIC10, we used *Da-gal4* to induce the overexpression of *14-3-3zeta* (Fig. S5, *C*–*E*), while *Da-gal4>W*^*1118*^ flies were used as the control group. When 40 μM TIC10 was applied to inhibit the AKT and ERK pathways, the results showed that the inhibitor TIC10 extended the lifespan of both *Da-gal4>14-3-3zeta* and *Da-gal4>W*^*1118*^ (the median lifespan extended 14 days and 9 days, respectively) (Fig. 6*B*; S5F). To determine whether the fly gut function was affected by the inhibitor TIC10, we examined gut intestinal barrier function, which is a good indicator of overall intestinal integrity. The proportion of “Smurfs” in the control population increased from Day 20 to Day 40 (Fig. 6*C*). Again, the addition of TIC10 significantly decreased the population of Smurfs on Day 20 in the 14-3-3zeta-overexpressing group, although the difference did not reach statistical significance at Day 40.

At the protein expression level, TIC10 treatment reduced the levels of pERK and pAKT in both *Da-gal4>14-3-3zeta* and *Da-gal4>W*^*1118*^ flies (Fig. 6, *D* and *E*). The results indicated that TIC10 can not only restore the lifespan of overexpressed *14-3-3zeta* flies to the normal level but also prolong the lifespan of control flies. Taken together, the inhibitor TIC10 can inactivate AKT and ERK to extend the lifespan of the fly.

We then examined whether the inhibitor TIC10 could affect intestinal immune homeostasis. When applying *ECC15* to infect *Drosophila*, we found that the addition of TIC10 significantly prolonged the lifespan of *ECC15*-infected flies in both the control and *14-3-3zeta* overexpressed groups (Fig. 6*F*), indicating that TIC10 treatment can improve the immune imbalance caused by 14-3-3zeta overexpression. TIC10 significantly downregulated the expression levels of the antimicrobial peptide genes *dpt* and *ATTA* caused by overexpression of 14-3-3zeta (Fig. 6, *G* and *H*), suggesting that TIC10 can rescue the shortened lifespan and immune disorder phenotype caused by overexpression of *14-3-3zeta*.

## Discussion

The 14-3-3zeta protein is a key member of the 14-3-3 protein family, known for its involvement in the regulation of neurodegenerative diseases (7). However, its role in the natural aging process remains largely unexplored. In this study, we have discovered that 14-3-3zeta plays a crucial role in the regulation of aging. As an individual age, the levels of 14-3-3zeta increase, and higher levels of this protein can accelerate the aging process, leading to intestinal immune disorders and a shorter lifespan in *Drosophila*. On the other hand, reducing the levels of 14-3-3zeta through RNAi significantly extends the lifespan of the fly. These findings suggest that 14-3-3zeta could be a potential target for interventions aiming to slow down the aging process.

### 14-3-3zeta may be a new antiaging target

The 14-3-3zeta is highly expressed in a variety of cancers, including breast, ovarian, prostate, lung, and stomach cancers (43). This high expression of 14-3-3zeta has been associated with poor prognosis and resistance to these cancers (but not limited to) (44). Our observation in the samples of fruit flies and mice at different ages also found that the protein levels of 14-3-3zeta increased significantly during senescence. In mice, transgenic 14-3-3zeta overexpression potentiates obesity (10). Loss of 14-3-3epsilon, another isoform of the 14-3-3 protein family in *Drosophila* and consequent chronic activation of FOXO resulted in smaller flies that lived longer, while overexpression of 14-3-3epsilon did not significantly affect the lifespan (15). In our results loss function of 14-3-3zeta, has significantly extended the lifespan (Fig. 4*A*), while overexpression of 14-3-3zeta significantly shorted the lifespan (Fig. 2*A*). Furthermore, silencing of *14-3-3epsilon* and *14-3-3zeta* in the larval salivary gland cells had differential phenotypes, showing isoform-specific functions of 14-3-3 proteins (14). In Hep-2 laryngeal cancer cells, RNA interference suppressed the expression of *14-3-3zeta*, inducing senescence *via* a p27-dependent pathway (45). *In vivo* experiments have also shown that both systemic and tissue-specific overexpression of *14-3-3zeta* can significantly shorten the lifespan of *Drosophila* and damage the integrity of the intestinal barrier (Fig. 2). The elevated expression of 14-3-3zeta is associated with high-risk myeloma genetic subtypes and a worse prognosis overall (46). In addition, recent studies by *Wan et al.* showed that *YWHAZ* (the gene encoding 14-3-3zeta protein) is a novel intellectual disability causative gene (47). All results implied that excessively high levels of 14-3-3zeta are not beneficial to the health of cells and animals. Therefore, it is necessary to accurately regulate the expression level of *14-3-3zeta* over the whole lifespan.

We observed changes in the abundance of phosphorylated forms of AKT, ERK, and FOXO in overexpression *14-3-3zeta* cells and flies (Figs. 5 and 6*D*). This is consistent with previous reports by other researchers on the role of 14-3-3zeta in the function of these proteins. Previous reports showed that 14-3-3zeta enhanced Akt phosphorylation through activation of phosphoinositide 3-kinase (PI3K) (21). 14-3-3zeta has also been confirmed to interact with RAF (48) and KSR (49) upstream of ERK to increase the phosphorylation level of ERK. 14-3-3zeta binds to pFOXO1, thereby promoting the nuclear extrusion of transcription factors. The 14-3-3zeta dimer can act as a partner to bind pFOXO to prevent its dephosphorylation, thereby promoting transcription factor extrusion from the nucleus (50). In addition, AKT kinase can directly phosphorylate FOXO1 (51). The effect of 14-3-3zeta on the transcriptional activity of FOXO was also confirmed by detecting the expression level of the target gene of transcription factor FOXO (Figs. 2 and 3). Since the regulatory roles of AKT, ERK, and FOXO in aging have been widely studied (52, 53, 54), we can think that 14-3-3zeta is an important link in the aging regulatory network.

Contrary to the gain-of-function results, the loss-of-function of 14-3-3zeta promoted the longevity of fruit flies. The expression of *Il-6* and *Il-8* could not be induced by the inflammatory cytokine interleukin-17A in *14-3-3zeta* ^*KO*^ ARPE-19 cells (55), which implies that 14-3-3zeta is necessary for the normal expression of inflammatory factors. Silencing the expression of *14-3-3zeta* can extend the lifespan of fruit flies. This suggested that limiting zeta protein to a reasonable expression level is beneficial to health and longevity. Thus, 14-3-3zeta can be used as a target for aging intervention.

### 14-3-3zeta affects intestinal immunity in aging

Previous work has demonstrated the complex relationship between bacterial load and lifespan, identifying both beneficial and detrimental effects of gut bacteria on flies (38, 56). *Drosophila* PGRP-SC1a/b and SC2 with amidase activity were shown to be responsible for the removal of peptides from glycan chains, thereby negatively regulating the IMD pathway (57). In our results, with the aggravation of aging fruit flies caused by overexpression of 14-3-3zeta, a significant decrease in the expression level of *pgrp-scs* was observed (Fig. 3). At the same time, we detected a significant increase in the expression levels of genes such as antimicrobial peptides *dpt* and *atta* downstream of the IMD signaling pathway. Previous studies have shown that high levels of antimicrobial peptide production contribute to aging through cytotoxic effects in *Drosophila* tissues (58), possibly one of the reasons for the shortened lifespan of flies overexpressing *14-3-3zeta*. Overexpression of *pgrp-sc2* increases pathogen clearance in *Drosophila* and reduces gut microbial load (38). When faced with *ECC15* infection, *14-3-3zeta* overexpressed flies have a lower ability to clear bacteria in a short time. The 14-3-3zeta has recently been reported to be involved in the innate immune response of Pacific abalone (*Haliotis discus hannai*) (59). The effector regulatory protein 14-3-3zeta is involved in *Legionella pneumophila* targeting the microtubule-associated protein Phldb2 to inhibit host cell migration (60). Combined with our findings, all outcomes imply that 14-3-3zeta has effects in regulating innate immunity. Therefore, it is suggested that 14-3-3zeta can modulate the expression of PGRPs, thereby altering the gene expression of antimicrobial peptides in intestinal tissues, as well as the ability to clear pathogenic microorganisms and reshape intestinal immunity in *Drosophila*. Given the important impact of immune homeostasis in aging, we speculate that this may be an important factor in the regulation of lifespan and aging by 14-3-3zeta.

### TIC10 recovered 14-3-3zeta overexpression-induced aging and may be a potential antiaging therapeutic

Numerous studies have explained in detail the role of the AKT and ERK pathways in aging and disease (25, 52, 61, 62). Previous studies have shown that TIC10 can inhibit the activity of AKT and ERK and promote FOXO3a translocation into the nucleus (27). As AKT and ERK signaling is the central effectors by which 14-3-3zeta induces aging, TIC10 which reduced the activity of AKT and ERK showed an excellent effect against a shortened lifespan of *14-3-3zeta* overexpression.

In addition to antagonizing the senescence of *14-3-3zeta* overexpression, we also noticed that TIC10 treatment could increase the lifespan of *Drosophila* in the control group, and prolong the lifespan after *ECC15* infection. This suggests that TIC10 may act as a potential antiaging drug.

Collectively, the results from our *in vivo* and *in vitro* models demonstrate that the limitation of the abnormal increase in 14-3-3zeta in the process of aging is an important approach to maintaining health and lifespan. More importantly, the AKT and ERK signaling pathways play an important role in the 14-3-3zeta network regulating aging. Therefore, inhibiting the activity of these two pathways can practically reverse the aging caused by overexpression of 14-3-3zeta. Our work explored the role of 14-3-3zeta in the aging process and established a related regulatory network, which provides a basis for using 14-3-3zeta as a target for intervention strategies in aging.

## Experimental procedures

### Fly stocks and husbandry

*Da*^*GS*^*-gal4* and *Da-gal4* were obtained from the Bloomington *Drosophila* Stock Center (flhttp://flystocks.bio.indiana.edu), *S106*, and *5966*^*GS*^*-gal4* were a kind gift from the Pankaj Kapahi laboratory, and *14-3-3zeta RNAi* (*Thu2964, Thu4850*) was obtained from the Tsinghua University fly stock center. The 14-3-3zeta-HA (F1064) was obtained from the FlyORF stock center, and the human 14-3-3zeta-V5 transgenic line is described in the transgenic fly section. All of the lines were backcrossed into *w*^*1118*^ for at least six generations. Males of RNAi or transgenic flies were crossed with *gal4* virgin females, and F1 generation females were collected and, after mating, transferred to food containing 200 μM RU486 (Mifepristone) for activation. All stocks were maintained at 25 °C on a 12 h:12 h light: dark cycle at constant humidity using SYA food (63). Drugs (RU486, TIC10) were added to the food after cooling to 50 °C. For all experiments, flies were reared at standard larval density, and enclosed adults were collected over 12 h. Flies were mated for 48 h before being sorted into single sexes.

### Mice

Wild-type (WT) C57BL/6J female mice at 7 weeks and 14 months were purchased from Biocytogen. Animals had free access to water and food. All experimental protocols involving animals were approved by the Institutional Animal Care and Use Committee.

### Antibodies and reagents

The primary antibodies used were as follows: α-tubulin antibody (Sigma T9026), pFOXO (Cell Signaling Technology, 9461S) for *Drosophila* pFOXO, rabbit anti-foxo laboratory made as (64) for detecting Foxo in *Drosophila*. pFOXO1（ImmunoWay, YP0113）for detecting human pFOXO1, FOXO (Abcam, ab52857) for detecting human FOXO1, pERK (Cell Signaling Technology, 9101); ERK (Cell Signaling Technology, 4695), AOP (Developmental Studies Hybridoma Bank, anti-yan-8b12h9); GAPDH (Service Biotechnology Co, Ltd, GB11002); pAKT (Cell Signaling Technology, 4060); TEL (ImmunoWay, YT4600） and AKT (Cell Signaling Technology, 9272). Mifepristone was purchased from Sigma (M8046 Sigma). TIC10 (S7963) was purchased from Selleck.

### RNA extraction, cDNA synthesis, and quantitative real-time PCR

Total RNA was extracted from 30-day-old whole flies. Samples were treated with DNase, and then cDNA synthesis was carried out using the First Strand cDNA Synthesis Kit from Fermentas. PCR was performed with PowerUP SYBR Green Master Mix (Ref#A25777, Applied Biosystems) on a Bio-Rad Real-Time PCR system. The cycling conditions were as follows: 95 °C for 10 min; 95 °C for 15 s; 60 °C for 60 s, cycled 40 times, and equivalent amplicons of Actin5C were used as a reference for normalization. Quantitative real-time polymerase chain reaction (qRT–PCR) was performed as described previously [26]. The primers are listed below.

**Table:** 

Gene	Primers
H-*ywhaz*	AGCTGGTTCAGAAGGCCAAA
	AAGATGACCTACGGGCTCCT
*pgrpsc1b*	CAGCTTCCTGGGCAACTACA
	GTACAGGATGTAGCCGGAGC
*14-3-3zeta*	ACATCCCATCCGTTTGGGTC
	TCGTTCAGTGTGTCCAGCTC
*pgrpsc2*	GGCGTGACCATCATCTCCAA
	AGTAGTTTCCAGCGGTGTGG
*pgrpsc1a*	CAGCTTCCTGGGCAACTACA
	GTACAGGATGTAGCCGGAGC
*CeCC*	CATTGGACAATCGGAAGCCG
	GCGCAATTCCCAGTCCTTGA
*Dpt*	GGCTTATCCGATGCCCGACG
	TCTGTAGGTGTAGGTGCTTCCC
*Atta*	TCGTTTGGATCTGACCAAGGGCAT
	TTCCGCTGGAACTCGAAACCATTG
*inr*	CATCGGAAGGGAGGCGTAA
	CGTTTGCCTAATCGTCGAACA
*4ebp*	ACCCTCTACTCCACCACTCC
	GGAGTTTGGCTCAATGGGGA
*Drosomycin*	CGTGAGAACCTTTTCCAATATGAT
	TCCCAGGACCACCAGCAT

### Lifespan analysis

Flies were collected when enclosed over 48 h and allowed to mate. Females were randomly allocated to the experimental food treatments and housed in plastic vials containing food at a density of 10 flies per vial, with 10 vials per condition (n = 100). Flies were transferred to the fresh food source 3 times per week, during which any deaths and censors were recorded. For lifespan assays, three independent experiments were carried out.

### Smurf assay

Unless stated otherwise, flies were aged on medium until the day of the Smurf assay. The dyed medium was prepared using standard media with dyes added at a concentration of 2.5% (wt/vol) Blue dye no. 1 (Sigma–Aldrich). Flies were maintained on a dyed medium for 9 h. A fly was counted as a Smurf when dye coloration could be observed outside of the digestive tract. To calculate the Smurf Increase Rate (SIR), we plotted the average proportion of Smurfs per vial as a function of chronological age and defined the SIR as the slope of the calculated regression line.

### Climbing assay

*Drosophila* was placed in a tube, which was gently shaken so that the fruit fly went into the bottom of the tube. The flies climbed freely for 45 s and were then photographed. The assay was repeated three times with independent groups of *Drosophila*. The speed was calculated by the formula: PI= (ntot+ntop-nbot)/2/ntot (ntot indicates all *Drosophila*, ntop indicates the *Drosophila* at the top of the tube, nbot indicates the *Drosophila* at the bottom of the tube, and PI stands for climbing ability).

### Stress index

Each group of experiments required 200 flies that had been fed for 10 days. For the paraquat assays, *Drosophila* was fed 20 mM paraquat diluted in a 5% glucose solution supplied on filter paper. In the starvation test, the *Drosophila* were placed in a tube containing 1.5% agarose to provide only moisture. The number of deaths was recorded every 0.5 days in the above experiments.

### Transfection

The day before transfection, 293T cells were seeded per 6-well plate in 1.5 ml of complete medium. The cells were incubated at 37 °C and 5%CO_2_. On the day of transfection, following the Effectene Transfection Reagent Handbook (QIAGEN), 0.4 μg of DNA was used per well. Effectene-DNA complexes were removed after 24 h, and a fresh complete medium was added. Follow-up experiments were carried out 12 to 24 h later.

### RNA sequencing

Flies were reared under controlled aging conditions for 30 days. Flies were immediately transferred to a tube on dry ice. Three biological replicates were used per condition. Notably, samples were generated independently. Total RNA was extracted using TRIzol (Invitrogen) according to the manufacturer’s instructions, including DNase treatment. Quality control was performed by an Experion Automated Electrophoresis System (Bio–Rad). Three biological replicates were used per condition. rRNA-depleted libraries were generated at the Max Planck Genome-Centre Cologne (Germany). RNA sequencing was performed with an Illumina HighSeq2500 and 37.5 million single-end reads/sample.

### Transgenic fly construction

Human 14-3-3zeta cDNA was amplified by Takara Primer STAR PCR enzyme from the 293T cell line; the primers used were as follows:

F: TTGCGGCCGCGCCACCATGGATAAAAATGAGCTGGTTC,

R: TGCTCTAGAATTTTCCCCTCCTTCTCCTGC. The cDNA of human *14-3-3zeta* was cloned into the pUAST-attB vectors. The resulting plasmids were injected into embryos of [*y1 Mvas-int. DmZH-2A w∗*; *M3xP3-RFP-attP ZH-86Fb*] flies (BDSC #24749). Individual males from the injected embryos were then crossed with flies containing the third chromosome balancers *TM6B*, *Tb/TM3*, *Sb* to establish balanced stocks.

### Western blotting

Total protein was isolated from female mouse tissues or fly bodies or cells in a buffer containing 20 mM Tris, pH 7.5, 1% Triton X-100, 1 mM EDTA, 1 mM EGTA, and protease/phosphatase inhibitors. Centrifuge the lysate and store 20 to 50 mg of the supernatant in Laemmli sample buffer (containing 62.5 mM Tris, 2% SDS, 25% glycerol, 0.01% bromophenol blue, and 5% β-mercaptoethanol). Proteins were incubated at 100 °C for 5 min and immunoblotted. Samples were separated by SDS-PAGE and transferred to polyvinylidene fluoride membranes. The membrane was blocked with 5% skim milk, 20 mM Tris, 500 mM sodium chloride, and 0.5% Tween-20 for 2 h and detected with primary antibody.

### Infection of adults by Ecc15

The monoclonal bacterium Ecc15 was cultured overnight by shaking at 200 rpm at 30 °C. Bacteria were collected after centrifuging at 6000*g* for 10 min at 4 °C and washed twice with sterile PBS at 7500*g* for 5 min at 4 °C. The bacteria were resuspended with sterile 5% sucrose solution as OD600 = 200. For oral infection, for 4 to 6 days female flies were starved for 2 h before being transferred in vials fully covered by two layers of filter paper containing 200 ml Ecc15. Adult flies were transferred to vials with fresh Ecc15 every 2 days.

### Quantification and statistical analysis

All statistical analyses were carried out using GraphPad Prism 8.00 software. Measurements represent the mean and error bars represent the standard deviation. As appropriate, Student’s unpaired 2-tailed *t* test or one-way ANOVA with post hoc Dunnett’s test was used to calculate significance. For all tests, *p* < 0.05 was considered significant. Asterisks reflecting the calculated *p* values are shown above each measurement, and ns indicates that differences between measurements were not statistically significant.

## Data availability

The RNA-seq dataset has been deposited in SRA, (Overexpression *14-3-3zeta* in *Drosophila*) under accession code PRJNA780618.

## Supporting information

This article contains supporting information.

## Conflict of interest

The authors declare that they have no conflicts of interest with the contents of this article.
