# Supplementary material for: The adaptor protein 14-3-3zeta modulates intestinal immunity and aging in Drosophila
Source: J Biol Chem. 2023 Oct 31;299(12):105414. doi: 10.1016/j.jbc.2023.105414 (PMC10724694; doi:10.1016/j.jbc.2023.105414)
Supplement: Supporting information [file mmc1.docx]

**Supporting Information**

**14-3-3zeta modulates intestinal immunity and aging in *Drosophila***

Xiaolan Fan^1,2#^, Tiantian Huang^1#^, Shuai Wang^1#^, Ziyue Yang^1^, Wenhao Song ^1^, Yao Zeng ^1,3^, Yingdong Tong^1^, Yujuan Cai^1^, Deying Yang^1,2^, Bo Zeng^1,2^, Mingwang Zhang^1,2^, Qingyong Ni^1,2^, Yan Li^1,2^, Diyan Li^1,2^, Mingyao Yang^1,2^ *

^1^Institute of Animal Genetics and Breeding, Sichuan Agricultural University, Chengdu, Sichuan, 611130, P. R. China

^2^Farm Animal Genetic Resources Exploration and Innovation Key Laboratory of Sichuan Province, Sichuan Agricultural University, Chengdu, Sichuan, China

^3^Technology Institute of Silk and Mulberry, ChongQing Academy of Animal Sciences, Rongchang, Chongqing, 402460, P. R. China

**Supplementary Figures**

**
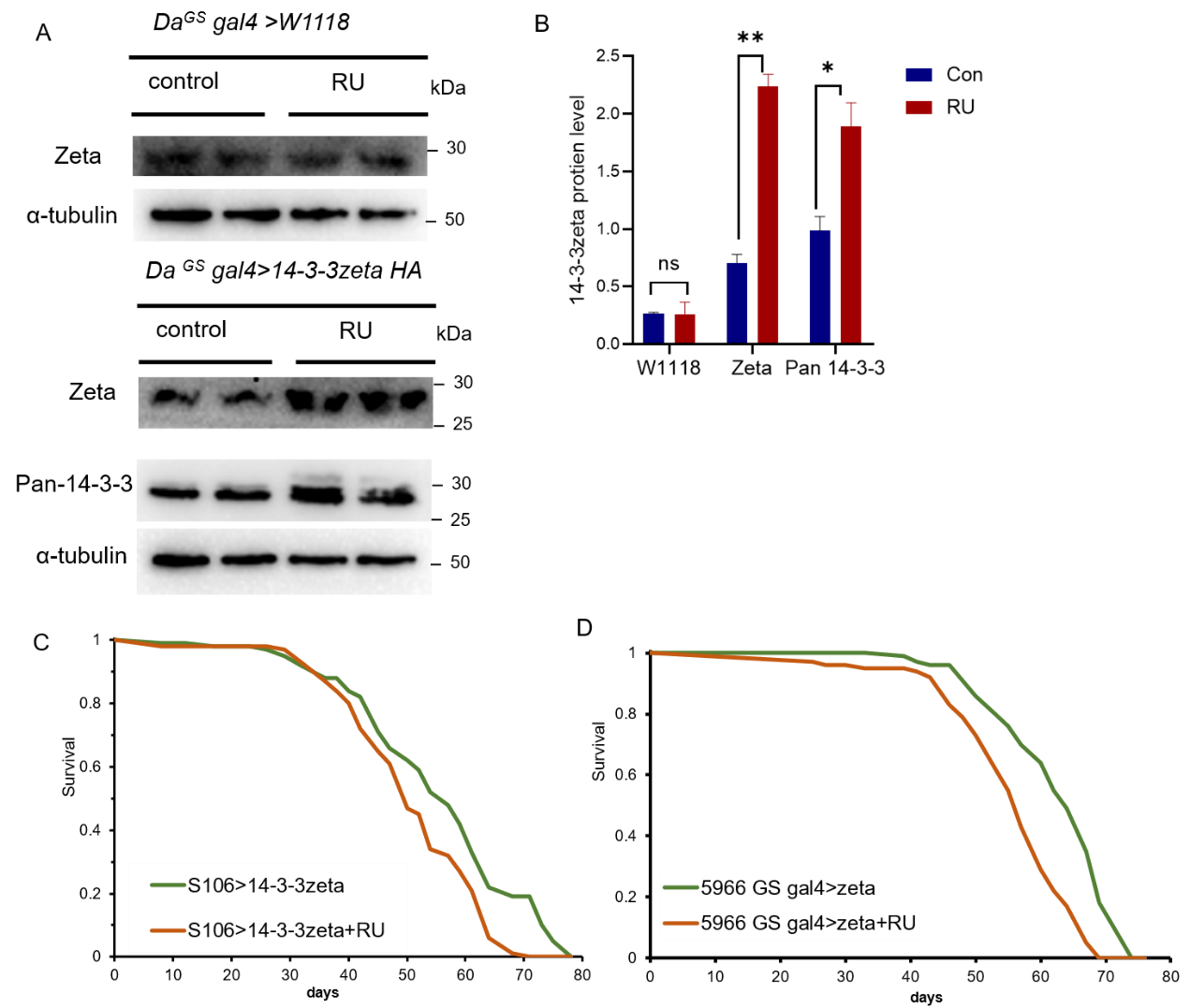
**

**Figure S1. The 14-3-3zeta participates the aging in *vivo***.

A, Flies with indicated genotypes were collected samples were then lysed and subjected to WB to examine the levels of indicated proteins. B, The bar graph illustrated the densitometry of the blots(right panel)，(Error bars indicate SD, ns p>0.05, **p<0.01,***p<0.001, n=2). C, The lifespan of flies was shortened significantly by overexpression of 14-3-3zeta using the gut and fat body specific S106-Gal4 driver(p<0.0001,n=100, log-rank test). D, The lifespan of flies was shortened significantly by overexpression of h14-3-3zeta using the 5966 ^GS^ Gal4 driver(p<0.0001,n=100, log-rank test).


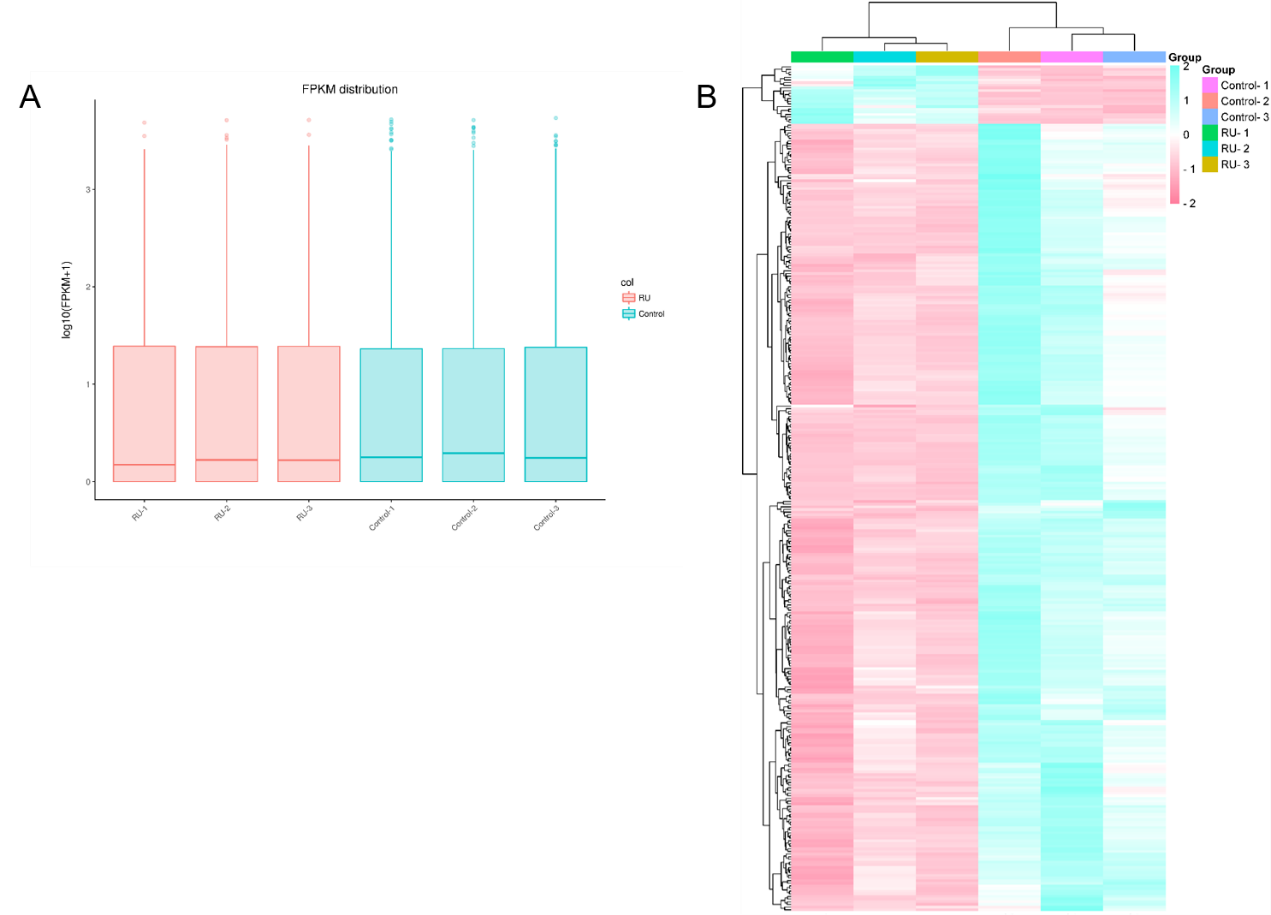


**Figure S2. The different expression genes of 14-3-3zeta overexpression.** A, Fragments per million per kilobase (FPKM) of 14-3-3zeta overexpression and control. B, the heatmap of different expression genes compare *14-3-3zeta* overexpression with the control group.


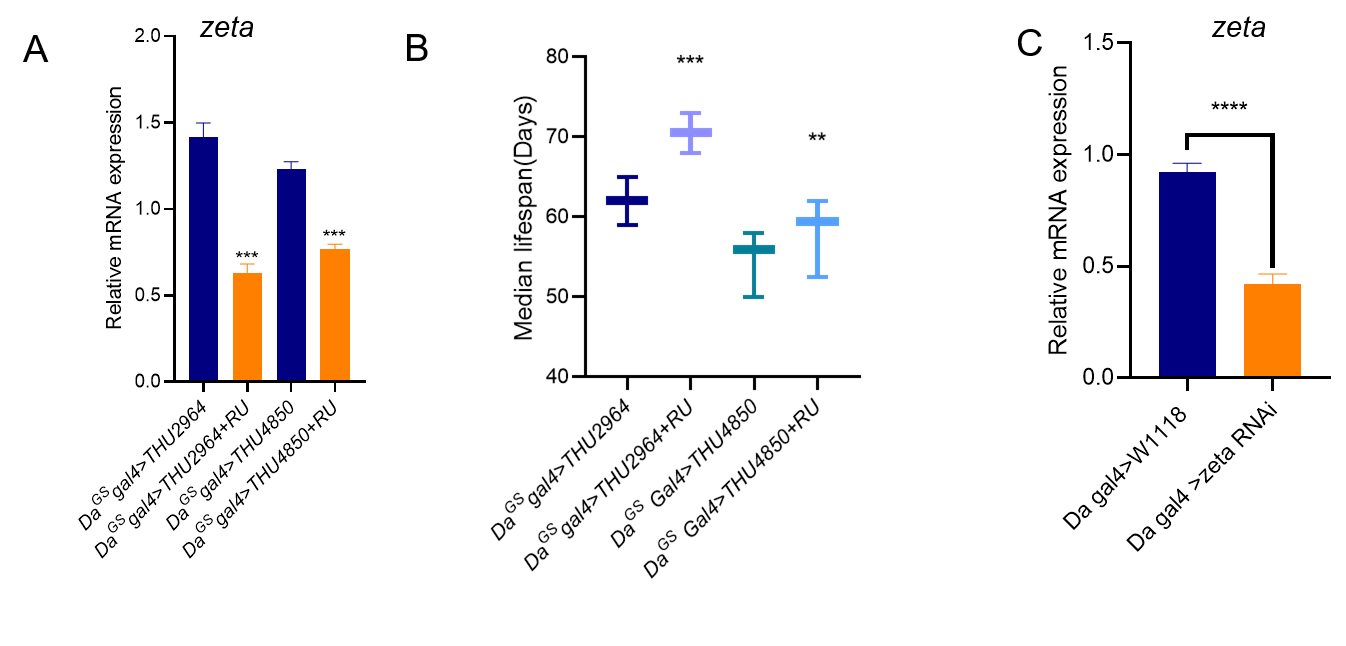


**Figure S3. The 14-3-3zeta participates the aging in *vivo***.

A, The 14-3-3 zeta mRNA level in *Da GS gal4* and RU induce14-3-3zeta RNAi . (Error bars indicate SD, ***p<0.001,****p<0.0001, n=3). B, The median lifespan of ubiquitous knockdown of 14-3-3 and treatment with RU for 3 repeat experiments ( the p value is the RU treatment group compared nontreatment group, Error bars indicate SD, **p<0.01,***p<0.001). C, The *14-3-3 zeta* mRNA level in *Da gal4>w1118* and Da gal4>14-3-3zeta RNAi. (Error bars indicate SD, ****p<0.0001, n=3)


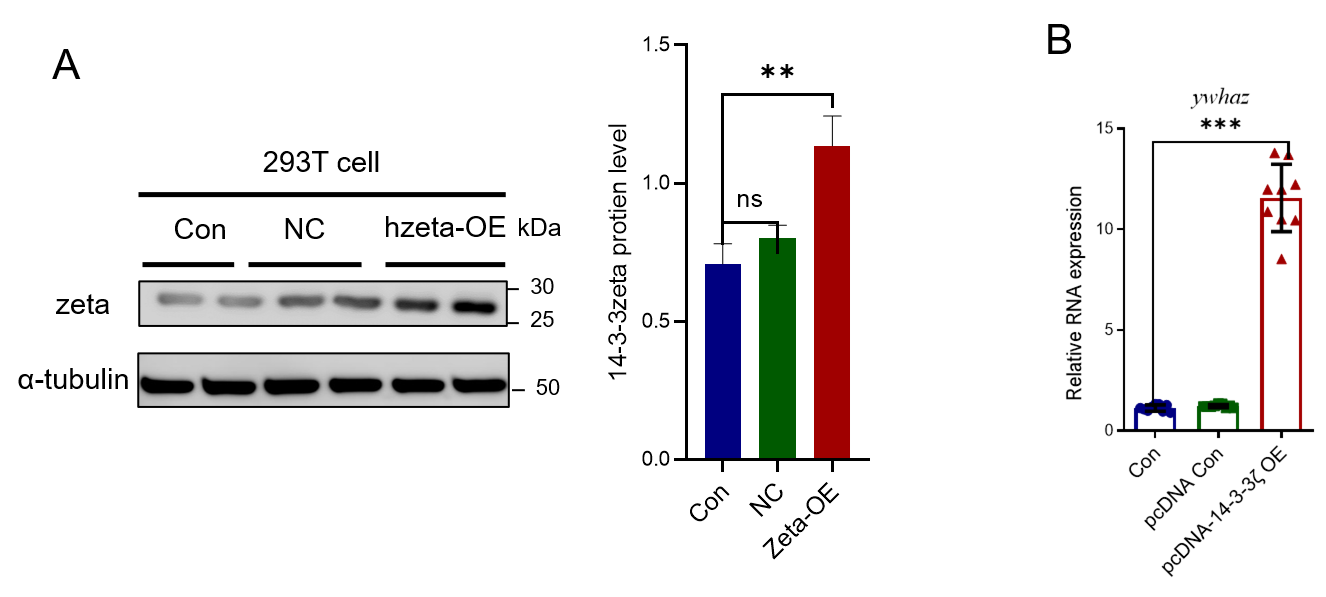


**Figure S4, The protein and mRNA expression level when the human14-3-3zeta overexpression in 293T cells.**

A, The 14-3-3 zeta protein level in overexpression h14-3-3zeta 293T cells（α-tubulin gel blots reused with the third row of figure 5E）. The bar graph illustrated the densitometry of the blots (Error bars indicate SD, ns: p>0.05, ** p<0.01, n=2). B, The 14-3-3 zeta mRNA level in overexpression *h14-3-3zeta* 293T cells. (Error bars indicate SD, ***p<0.001, n=3).


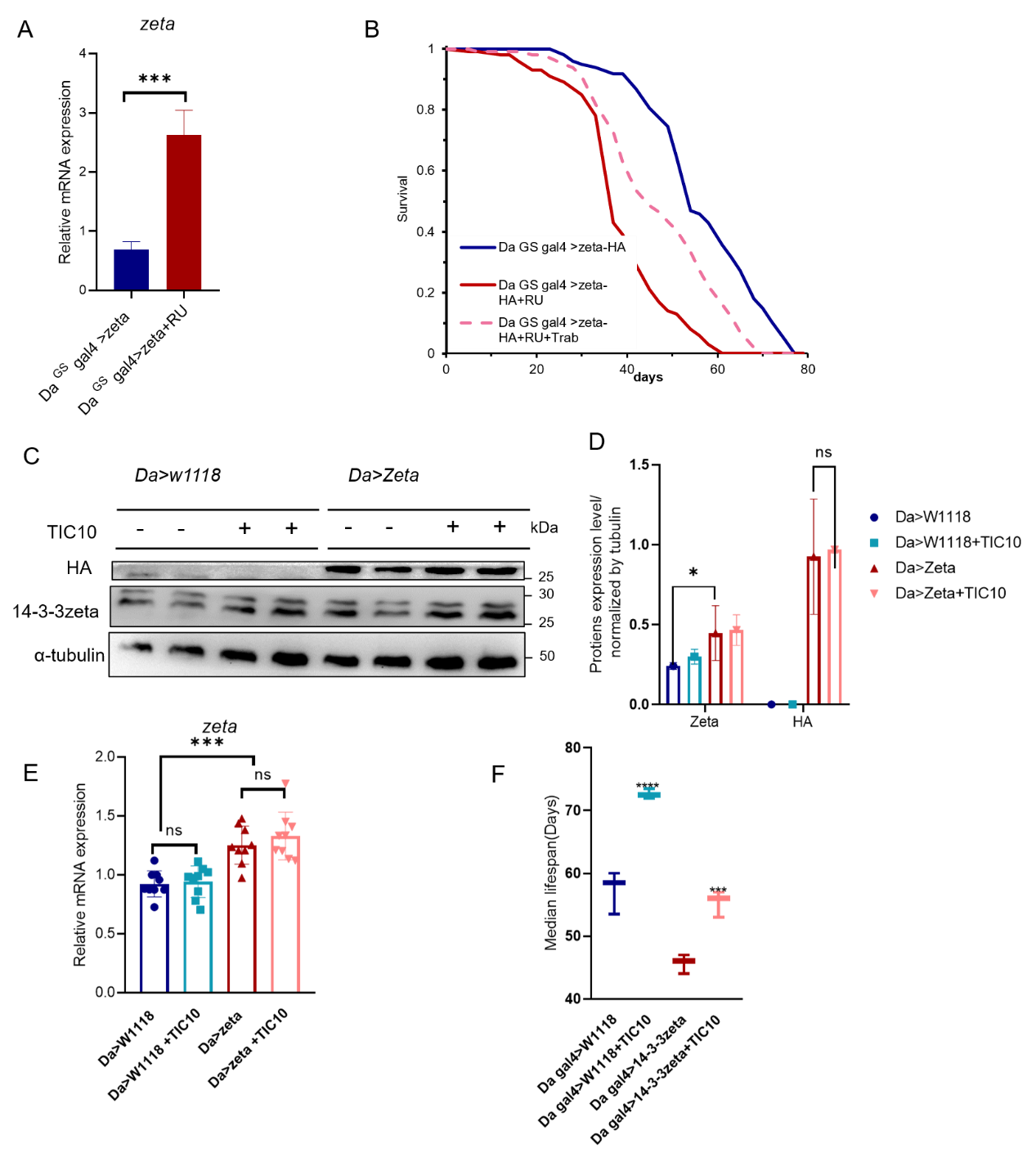


**Figure S5, the drugs restored the overexpression 14-3-3zeta shortened lifespan.**

A, The mRNA expression level of the RU untreated or treated the flies on the genotype of *Da^GS^gal4>14-3-3zeta* (Error bars indicate SD, ***p<0.001, n=3). B, The lifespan of the ERK inhibitor Trab treatment on the base of overexpression of *14-3-3zeta* ( the p-value is the Trab treatment group compared the *Da^GS^gal4>14-3-3zeta*+RU group p<0.0001,n=100, log-rank test). C-D, The protein level in flies with indicated genotypes and treatment. The bar graph illustrated the densitometry of the blots (Error bars indicate SD, ns p>0.05, * p<0.05, n=2). E, The mRNA expression level of the TIC10 treatment of the flies on the genotype of *Da gal4>W118* and overexpression *14-3-3zeta* (Error bars indicate SD, ns p>0.05, ***p<0.001, n=3). F, The median lifespan of the TIC10 treatment flies on the genotype of *Da gal4>W118* and overexpression *14-3-3zeta* for 3 repeat experiments (the p-value is the TIC10 treatment group compared the no TIC10 treatment group, Error bars indicate SD, ***p<0.001, ****p<0.0001, n=3).
